# Early Category-Specific Cortical Activation Revealed by Visual Stimulus Inversion

**DOI:** 10.1371/journal.pone.0003503

**Published:** 2008-10-23

**Authors:** Hanneke K. M. Meeren, Nouchine Hadjikhani, Seppo P. Ahlfors, Matti S. Hämäläinen, Beatrice de Gelder

**Affiliations:** 1 Cognitive and Affective Neuroscience Laboratory, Tilburg University, Tilburg, The Netherlands; 2 MGH/MIT/HMS Athinoula A. Martinos Center for Biomedical Imaging, Charlestown, Massachusetts, United States of America; 3 Harvard-MIT Health Sciences and Technology, Cambridge, Massachusetts, United States of America; 4 Brain Mind Institute, EPFL, Lausanne, Switzerland; University of Southern California, United States of America

## Abstract

Visual categorization may already start within the first 100-ms after stimulus onset, in contrast with the long-held view that during this early stage all complex stimuli are processed equally and that category-specific cortical activation occurs only at later stages. The neural basis of this proposed early stage of high-level analysis is however poorly understood. To address this question we used magnetoencephalography and anatomically-constrained distributed source modeling to monitor brain activity with millisecond-resolution while subjects performed an orientation task on the upright and upside-down presented images of three different stimulus categories: faces, houses and bodies. Significant inversion effects were found for all three stimulus categories between 70–100-ms after picture onset with a highly category-specific cortical distribution. Differential responses between upright and inverted faces were found in well-established face-selective areas of the inferior occipital cortex and right fusiform gyrus. In addition, early category-specific inversion effects were found well beyond visual areas. Our results provide the first direct evidence that category-specific processing in high-level category-sensitive cortical areas already takes place within the first 100-ms of visual processing, significantly earlier than previously thought, and suggests the existence of fast category-specific neocortical routes in the human brain.

## Introduction

We have the remarkable ability to recognize thousands of visual objects in our daily environment, such as faces, bodies, cars, keys, shoes, animals, food, tools, and houses. Despite its complexity, visual categorization is executed rapidly and effortlessly by the human brain. These computations appear to be mainly carried out by the ventral visual pathway [1], [2], through neurons with increasingly larger receptive fields, responding to increasingly complex features of the stimuli as one moves up within the hierarchy. The physical features of the input image are generally assumed to be first extracted in lower-level cortical areas (i.e., V1, V2, V4) [3], [4] before they are projected to higher-level regions in the occipito-temporal cortex where complex patterns are processed [5]–[8] and a visual representation of the input image is formed [9].

Functional neuroimaging studies (e.g. positron emission tomography, functional magnetic resonance imaging (fMRI)) in humans have examined the higher-level cortical regions involved in the visual perception of different objects. Faces [10]–[14], bodies [15]–[17], animals [18], [19], houses [20]–[22], tools [18], [19] and letter strings [23]–[25] have been shown to selectively activate focal regions of cortex. While the location of areas involved in object processing has been widely studied, the sequence and timing of activation of these areas is less well known. The long-held general assumption is that at least during the first 100-ms complex visual stimuli are generally processed in the same low-level areas [26], and that category-specific cortical activation occurs at later stages.

For instance, intracranial recordings in patients have shown that during face perception the well-established face-selective area of the fusiform gyrus becomes strongly activated around 170-ms after stimulus onset [24], [26]–[28]. This time course is corroborated by a prominent face-selective component around 170-ms [29] in recordings of electrical (EEG) and magnetic (MEG) brain activity from the scalp in healthy volunteers, labeled the ‘N170’ [30] in EEG studies or ‘M170’ in MEG recordings.

However, this traditional model of object perception is challenged by recent psychophysical and electrophysiological findings suggesting that visual categorization processes may already take place at even earlier latencies [31]–[35], i.e. around 100-ms post stimulus onset. Thorpe and colleagues [32], [35] found evidence for rapid visual categorization (the detection of animals versus non-animals in natural images) taking place within the first 100–150-ms after stimulus onset. In addition, category-specificity has also been claimed for an earlier component that peaks around 100–120-ms after the onset of a visual stimulus in posterior sensors in EEG or MEG recordings, labeled the ‘P1’ and ‘M100’ component respectively, or of even earlier activity (30–110-ms post-stimulus). Most of these interpretations are however heavily debated, as they were either based on inter-categorical comparisons [36]–[39] which suffer from serious low-level confounds [40], or on old-novel distinctions which may signal general repetition effects rather than face-recognition per se [41]–[44]. More convincing evidence for rapid face categorization was nevertheless provided by two studies free from low-level stimulus confounds. Liu and colleagues [33] found that the M100 component is sensitive to the successful detection of faces embedded in noise. In addition, Debruille et al. found early differential responses between carefully matched photographs of known and unknown faces around 100-ms at frontocentral and centroparietal sites [45].

The neuronal underpinnings of this proposed early phase of visual categorization analysis remain however a puzzle. Reports on the neuronal origin of the P1/M100 response to faces have been inconsistent, as sources have been found in the retinotopic cortex of the medial occipital cortex [36], [46], posterior extrastriate cortex [47], [48], but also in high-level visual cortex of the mid-fusiform gyrus [47].

We took advantage of the high temporal resolution of magnetoencephalography (MEG) combined with its relatively good spatial resolution to investigate whether category-specific cortical activation may already take place within the first 100-ms. Early visual electrophysiological responses are known to be extremely sensitive to the physical attributes of the stimulus [40]. To avoid these low-level visual confounds we did not contrast the responses to different stimulus categories directly, but instead examined inversion effects, by comparing the differential responses between the upright and inverted presentation of three different stimulus categories, faces, bodies and houses. While physical stimulus properties remain unchanged, this simple procedure of stimulus inversion induces a large shift in the way some object classes are perceived, a phenomenon known as the inversion effect [49]. This is presumably due to the fact that presenting stimuli in non-canonical orientation interferes with the normal configural processing [50].

For the present experiment, we selected faces as a stimulus category because they show a strong behavioral [49]–[53] and electrophysiological inversion effect, and the face-sensitive cortical areas have been carefully mapped out. EEG and MEG recordings have yielded a robust neurological correlate of the face inversion effect, namely a delay and enhancement of the N170/M170 component [30], [46], [48], [54]–[58]. It is therefore commonly assumed that the extraction of the overall stimulus configuration takes place during this stage. However, consistent with the afore mentioned evidence of categorization taking place around 100-ms after stimulus onset [32], [33], there is now a growing number of observations of an earlier electrophysiological inversion effect occurring around 100–120-ms after picture onset for the P1/M100 component [48], [57], [59].

Bodies were chosen as a second type of biologically salient stimuli with strong configurational properties and a special perceptual status somewhat similar to that of faces [60]–[63], similar electrophysiological correlates like the elicitation of an N170 component [61], [64]–[66] and an N170 inversion effect [64] and a partially common neuro-anatomical substrate [16], [67]–[69]. Houses were used as an example of a non-biological stimulus class with a clear canonical orientation but without a strong behavioral inversion effect [50], [63].

**Figure 1:**
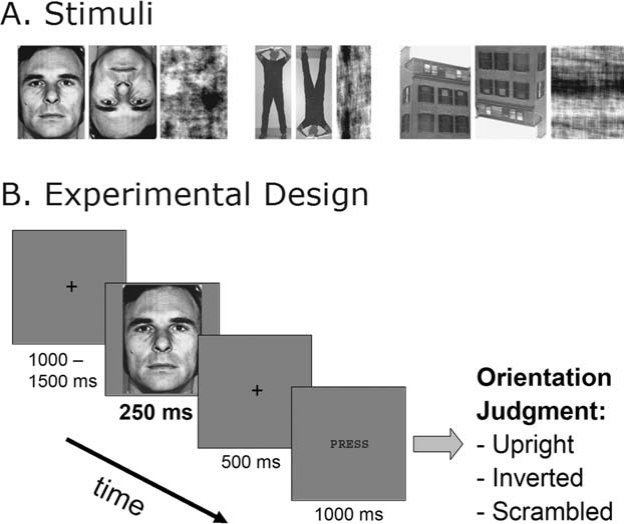
Examples of Stimuli and Experimental Trial. A. Examples of the nine stimulus conditions. Photographs of Faces, Bodies and Houses were presented in three different ways: Upright, Inverted, and after phase-Scrambling. B. Example of an experimental trial. Stimuli were presented for 250-ms in random order, and after a delay of 500-ms subjects had to judge by button press whether the pictures were Upright, Inverted, or Scrambled.

Participants viewed photographs of faces, bodies and houses presented in either their upright or inverted orientation or in a Fourier phase-scrambled version, and were asked to classify them accordingly, i.e. as upright, inverted or scrambled (see Figure 1 for examples of stimuli and the experimental paradigm). We used magnetoencephalography and anatomically-constrained distributed source modeling [70] to monitor brain activity with millisecond-resolution in order to examine early category-specific cortical activity related to the inversion effects during the M100 stage of visual processing. Our results show that category-specific processing in high-level category-sensitive cortical areas already occurs during the first 100-ms of visual processing, much earlier than previously thought, hereby shedding a new light on the early neural mechanisms of visual object processing.

## Results

### General Description of Evoked Responses

**Figure 2:**
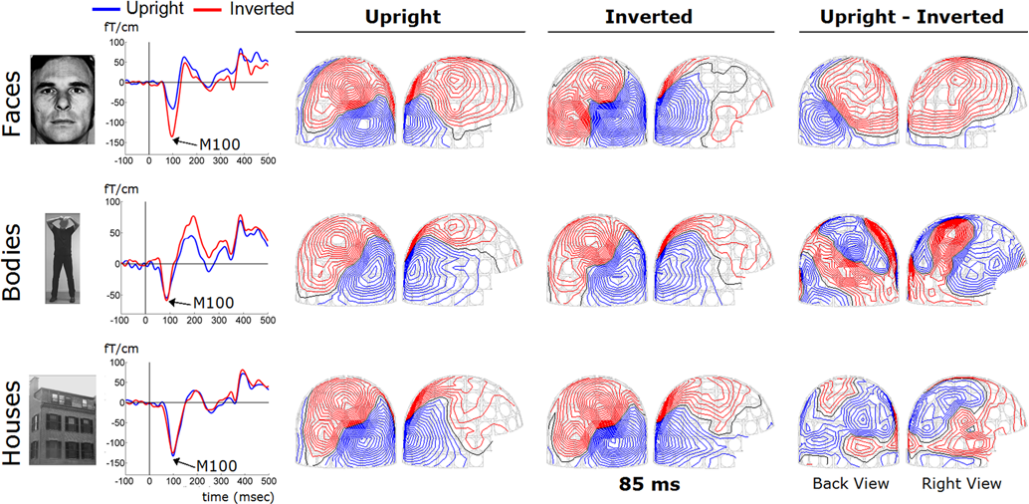
Visually evoked magnetic fields to Upright and Inverted Stimuli. Visually evoked magnetic fields to Upright (blue) and Inverted (red) Faces, Bodies, and Houses recorded at a typical posterior planar gradiometer (MEG2123) in a representative individual. The early response peaked around ∼100-ms after picture onset, and is clearly smaller for Upright Faces as compared to Inverted Faces. Note the different vertical scale for Bodies as compared to Faces and Houses. Displayed on the right are the corresponding topographic distributions of the evoked fields at 85-ms latency for the Upright and Inverted conditions and for the Upright-Inverted difference-wave, as seen from the back and the right side of the helmet.

The event related magnetic fields to Faces showed a temporal and spatial distribution consistent with previous reports. The earliest prominent responses were maximal over midline occipital gradiometers; they started to rise around 45–60-ms and peaked around 80–100-ms, corresponding to the M100 component (Figure 2). This was rapidly followed by responses over more lateral occipito-temporal sensors peaking between 90–180-ms. The midline M100 occipital component was smaller for Upright Faces than for Inverted Faces. The responses to Bodies and Houses showed a spatiotemporal pattern that was similar to that of Faces during the first 100-ms. The field topography was already quite complicated during the rising phase of the M100 component for all three stimulus categories (Figure 2), suggesting a more complex configuration of underlying generators than a single dipolar source in the medial occipital cortex.

### Global Measures of Inversion Effects

**Figure 3:**
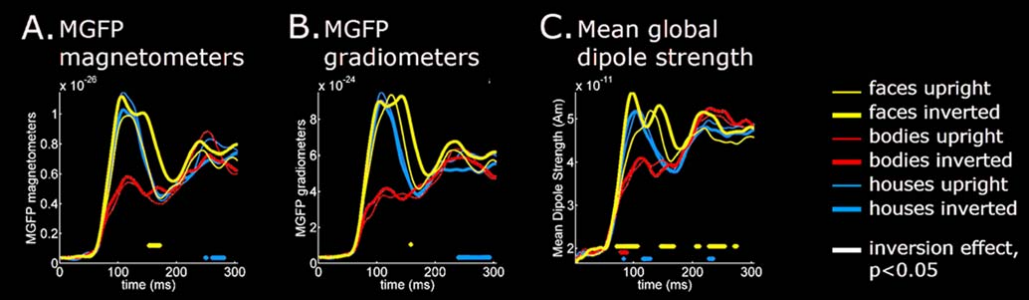
Global measures of MEG activity. A, B. The grand average (*n* = 9 subjects) of the Mean Global Field Power (MGFP) of the magnetometers (A) and gradiometers (B) showed a significant Inversion Effect for Faces only, i.e. around 170 ms. C. Source analysis (anatomically constrained MNE) revealed an early global Inversion Effect around 85-ms latency for all three stimulus categories with a larger mean overall ( = whole cortex) dipole strength for the Inverted stimuli than for the Upright stimuli. The time samples at which significant Inversion Effects occur (*p*<0.05; paired *t*-tests, *n* = 9, *df* = 8) are indicated by horizontal bars with color corresponding to category (Faces = yellow; Bodies = red; Houses = blue).

To quantify the data we first calculated the grand mean time courses of two global measures of the magnetic response across subjects: the mean global field power (MGFP) for the magnetometers and gradiometers and the mean global dipole strength over the cortex (Figure 3). When looking at the overall signal magnitude at the sensor level, the earliest Inversion Effect encountered in the MGFP was that for Faces around 160-ms (Figure 3a, b). Analysis of the global signals at the source level using the mean dipole strength across the whole brain, however, revealed Inversion Effects in all stimulus categories within the first 100-ms after stimulus onset, with stronger signals for the Inverted than for the Upright presentation (Figure 3c).

### Cortical Source Distribution of the M100 Inversion Effect

**Figure 4:**
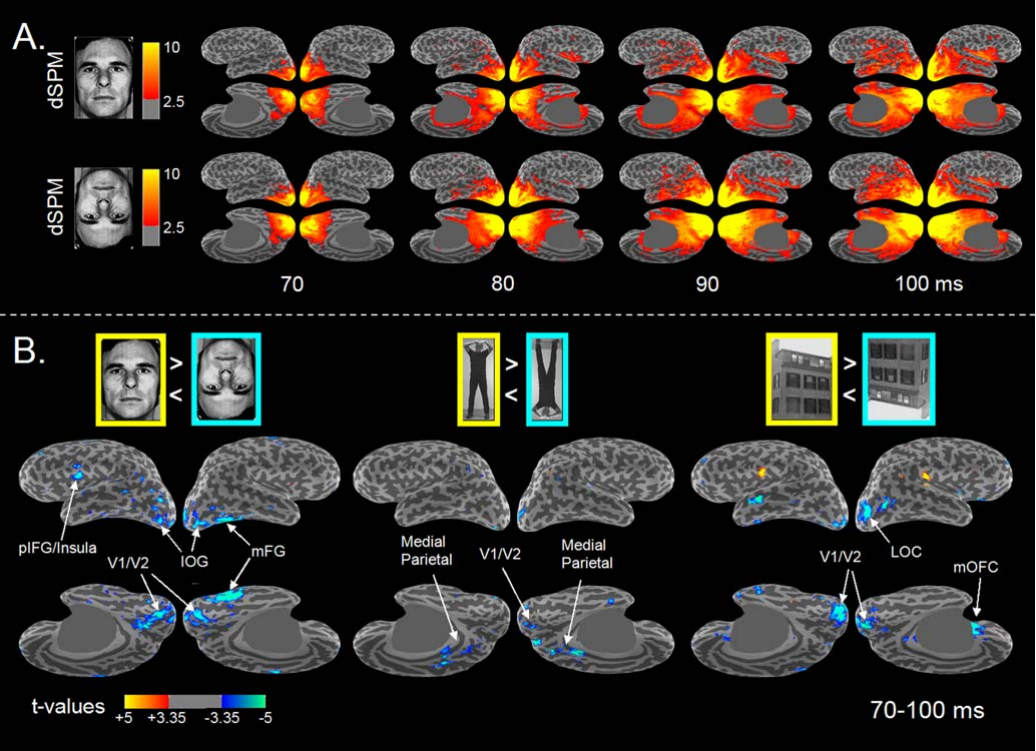
Source distribution of the M100 Stimulus Inversion Effect. A. Anatomically constrained source analysis (average dSPM values across subjects; *n* = 9) for Upright (top trace) and Inverted Faces (bottom trace) from 70–100-ms after stimulus onset visualized on the inflated cortical surface (gyri appear in light grey, sulci in dark grey). For each time-instant, four different views are presented to depict the whole cortical surface, with left hemisphere on the left and right hemisphere on the right of each quadruplet. The two top images of each quadruplet show the lateral aspects of the brain and a little strip of the ventral aspect (lateral view, 11° tilted towards the bottom view); the two bottom images show the medial and ventral aspects of the brain (medial view, 45° tilted towards bottom view). Only values of dSPM>2.5 are visualized. A grey opaque mask was placed over the midbrain. B. Differential activation related to the Inversion Effects, i.e. the contrast between the Upright and Inverted condition of Faces, Bodies and Houses. Displayed are the largest positive or negative t-values (two-tailed paired *t*-tests; *n* = 9; *df* = 8) at each dipole location occurring within the 70–100-ms time-window. Significant t-values at the level of *p*<0.01 are thresholded with respect to baseline noise and visualized only if the dipole strength exceeds a signal-to-noise ratio of 2.5 (i.e. dSPM>2.5) in at least one of the single stimulus conditions. The red and yellow colors denote locations at which the dipole strength is stronger for Upright than for Inverted stimuli. Blue colors denote locations in which the dipole strength is stronger for the Inverted stimuli. Absolute *t*-values of 3.35 and larger (red/dark-blue) correspond to *p*<0.01, absolute *t*-values of 4.8 and larger (yellow/light blue) to *p*<0.001. Abbreviations: mFG = middle Fusiform Gyrus; IOG = Inferior Occipital Gyrus; LOC = Lateral Occipital Cortex; pIFG = posterior Inferior Frontal Gyrus; mOFC = medial OrbitoFrontal Cortex.

Dynamic Statistical Parametric Maps (dSPM, [70]) confirmed that activation started focally in the medial surface of the occipital pole around 50–60-ms and spread out rapidly to more anterior, inferior and lateral areas, invading the ventral aspect of the temporal lobe within 100-ms (Figure 4a), with all stimulus categories showing roughly the same pattern. Cortical maps of the *t*-statistics for the contrasts between the Upright and Inverted conditions (Figure 4b), however, revealed that the M100 Inversion Effect had different cortical source distributions for the different categories.

**Figure 5:**
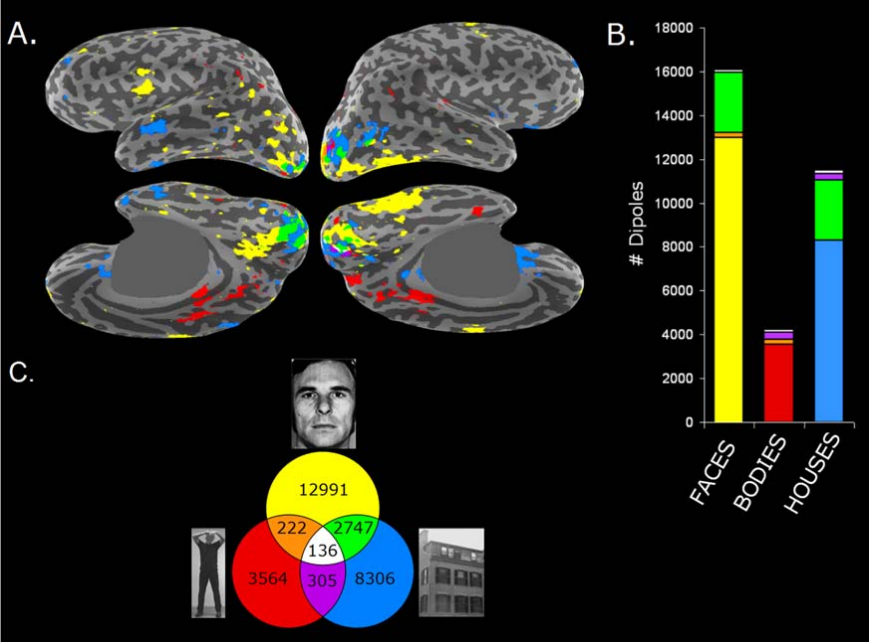
Category-specific cortical distribution of the M100 Inversion Effect. A. Overview of the cortical distribution of the Inversion Effect for the M100 component for the three different stimulus categories. The three *t*-maps of figure 4 were combined into a single map. Dipole positions at which the Inverted stimuli induced significantly (*p*<0.01) larger currents than their canonical Upright presentation within the 70–100-ms time-window are color-coded according to stimulus category and their spatial overlap as displayed in panel C. B. Bar graph displaying the number of dipoles showing a significant Inversion Effect for Faces, Bodies and Houses. Within each stimulus category the color (color coding as in C) indicates the degree of category-specificity, i.e. the amount of spatial (non-)overlap with the other categories. The amount of spatial overlap between categories is small (see also panel C for the exact number of dipoles). C. Color coding of stimulus category and their spatial overlap as used in panels A and B, with the exact number of dipoles showing a significant Inversion Effect.

Interestingly, apart from a general Inversion Effect in medial occipital cortex (∼area V1/V2) there was relatively little cortical overlap between categories (Figure 5a, 5b). Quantitative numbers for the amount of spatial overlap (Figure 5b) revealed that of all the 28,576 dipoles that showed significant inversion effects, 87% (24,681) did so for one category only, 12.5% (3,579) for two categories, and 0.5% (136) for all three categories. Besides substantial overlap between Faces and Houses (2,747 or 9.6%) which was found mainly in V1/V2 and the lateral occipital cortex (LOC), only negligible overlap was found for the other combinations of two or three categories. Hence, the number of dipoles with a category-specific M100 Inversion Effect by far outnumbered the number of overlap dipoles. Category-specific Inversion Effects for Faces (yellow) were found in the left and right inferior occipital gyrus (IOG), in the right middle fusiform gyrus (mFG) and in the transition area between the left posterior Inferior Frontal Gyrus (pars opercularis; pIFG) and Insula. In addition scattered clusters were found in the lateral occipital cortex and the lateral and inferior temporal lobe. The early Body Inversion Effect (red) was mainly found in posterio-dorsal medial parietal areas (the precuneus / posterior cingulate). For Houses (blue) large clusters were found in the right LOC, left anterior superior temporal sulcus and the right medial orbitofrontal region.

**Figure 6:**
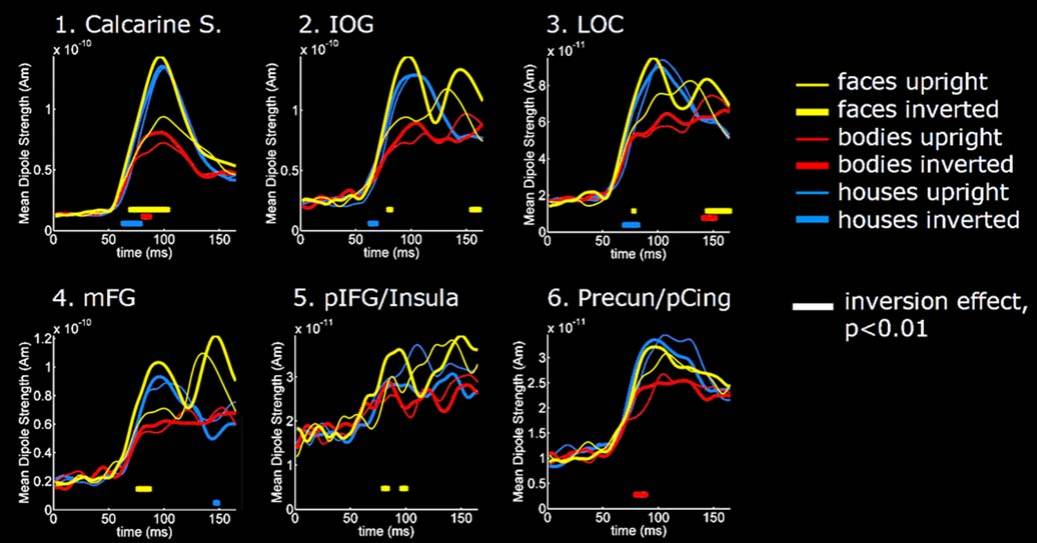
Time courses of current strength from selected regions. Grand average (*n* = 9 subjects) time courses of estimated currents extracted from six regions that showed significant Inversion Effects indicated on the inflated brains in the top panel, i.e. the Calcarine Sulcus (1), IOG (2), LOC (3), mFG (4), pIFG/Insula (5), and the Precuneus/posterior Cingulate (6) for Upright and Inverted Faces (yellow), Bodies (red) and Houses (blue). The time instants at which significant differences occur between Upright and Inverted conditions are indicated at the level of *p*<0.01 (paired *t*-tests, *n* = 9, *df* = 8) by horizontal bars with color corresponding to category. Abbreviations: Calcarine S. = Calcarine Sulcus; IOG = Inferior Occipital Gyrus; LOC = Lateral Occipital Cortex; mFG = mid-Fusiform Gyrus; pIFG = posterior Inferior Frontal Gyrus; Precun = Precuneus; pCing = posterior Cingulate Gyrus.

Extraction of the time-courses of the estimated currents from the six main regions that showed a significant Inversion Effect (Figure 6) confirmed that all stimulus categories evoked Inversion Effects in the right medial occipital cortex within the first 100-ms. For Faces and Bodies this was caused by a difference in signal magnitude that started at 70 and 82-ms post stimulus onset respectively. Upright and Inverted Houses however elicited equal peak amplitudes; the observed House Inversion Effect was due to a steeper rising phase of the M100 response for the Inverted images that already started to deviate from the response to Upright images at 63-ms. The same qualitative profile was found for the House Inversion Effects in the IOG (onset 57-ms) and LOC (onset 70-ms) of the right hemisphere. This was in contrast to the early Face Inversion Effects in IOG, LOC, mFG and pIFG/Insula that were caused by clear differences in peak amplitudes, with larger amplitudes for the Inverted Faces starting to significantly deviate from Upright Faces at 80, 78, 77 and 80-ms post stimulus onset respectively. The Body Inversion Effect in posterio-dorsal medial parietal areas (Precuneus / posterior cingulate gyrus) appeared to be caused by a transient drop in current strength for Upright Bodies between 80–93-ms.

## Discussion

### Summary of Main Results

We investigated whether category-specific cortical activation in the human brain already takes place within the first 100-ms of visual processing by comparing the MEG inversion effects of three stimulus categories: faces, bodies, and houses. We found that the first prominent MEG component peaking around 100-ms after stimulus onset (M100) was already sensitive to stimulus inversion of all three investigated object classes (Figure 3c). Significant inversion effects were found during the rising phase of the M100 between 70–100-ms post-stimulus onset, with larger responses for the inverted stimuli than for the upright stimuli. Distributed source analysis revealed that the cortical distribution of this early inversion effect was highly category-specific (Figures 4b, 5). Apart from the midline occipital cortex and the right lateral occipital cortex virtually no overlap between categories was found. Early face-selective inversion effects were found in areas that are part of the well-established distributed network for face-processing [12], [71], [72]: the inferior occipital gyrus (IOG), the right fusiform gyrus (FG), and the left posterior inferior frontal gyrus (pIFG, pars opercularis). For bodies early differential signals were found in the posterio-dorsal medial parietal areas (the precuneus / posterior cingulate). For houses the early inversion effect manifested itself mainly in the right lateral occipital cortex (LOC), and the right medial orbitofrontal cortex. Hence, our results show that different object categories already activate highly selective networks of neocortical areas within the first 100-ms after stimulus onset.

Importantly, category-specific cortical activation was identified on the basis of intra-categorical comparisons, i.e., we compared the responses to the upright stimuli with the responses to the same images when presented upside-down. As such we attempted to avoid confounds associated with the low-level physical properties of the stimuli. A nonspecific general inversion effect was found in the retinotopic areas of the medial occipital lobe (∼V1/V2), and an overlap between the face and house inversion effect was encountered in the right lateral occipital cortex. Although we cannot rule out that the effects in retinotopic areas may partly be caused by systematic low-level differences between the upper and bottom half of the stimuli, three important findings count in favor of category-specific processing. First, category-specific inversion effects were found well beyond retinotopic areas, even in prefrontal areas such as the posterior IFG and the medial orbitofrontal area. Second, the areas sensitive to stimulus inversion showed a strong hemispheric lateralization. Third, the cortical distribution of the face inversion effect was exactly found in those areas that are known to exhibit face-selective responses and that are part of the well-established distributed network for face processing (e.g. [12], [71], [72]): the inferior occipital cortex, the right middle fusiform gyrus, and the posterior inferior frontal gyrus.

### Comparison with Other Studies

The current result of an early inversion effect is consistent with previous MEG findings of an inversion effect for the M100–120 component [46], [48]. The M100 component has been demonstrated to be sensitive to the process of basic-level categorization, e.g. categorizing a face-stimulus as a face [33]. As such its sensitivity to stimulus inversion corroborates recent behavioral data showing that inversion causes an impairment in basic-level categorization [73], besides the well-known impairment in the recognition of facial identity and facial expression [50], [52], [53].

The currently observed location of the M100 inversion effect which indicates the early activation of a distributed network, is however in contrast with previous source analysis studies of the M100–120 face inversion effect [46], [48]. These previous studies either suggested only one sensitive brain area involved, i.e., a lateral occipital source [48], or were unable to demonstrate an inversion effect at the source level despite its presence at the sensor level [46]. This discrepancy may be explained by the different types of source localization methods applied. Whereas the previous studies were in principle based on localizing single point sources in the brain that can explain the measured magnetic fields, the present study applied a distributed source model, the cortically-based minimum norm estimate, which is well suited to analyze sources in an extensive network of brain areas that are activated more or less simultaneously [70]. The present results however correspond better with direct recordings from the human and monkey cortex which show that already within the first 100–120-ms after stimulus onset a multitude of brain areas are activated, even extending beyond visual areas [71], [74]–[76].

The general inversion effect found in the medial occipital cortex may be interpreted as the consequence of systematic differences in low-level properties (e.g., luminance for houses or local contrast for faces) between the upper and bottom halves of the image and the reported asymmetry for lower half-field and upper half-field VEF responses [77]. However, given the growing body of evidence that neurons in primary and secondary visual cortex (V1 and V2) can perform some kind of higher-level processing and are sensitive to stimulus features in natural scenes [e.g., 78], [79], we cannot rule out the possibility that some degree of higher-level sensitivity to stimulus orientation is already present in V1/V2. Moreover, recent evidence suggests the existence of even earlier neural encoding mechanisms of shape recognition already at the level of the retina [80].

The early MEG component in the fusiform gyrus corresponds to the initial potential around 90–110-ms recorded with intracerebral electrodes from the fusiform gyrus in human epilepsy patients [26], [71] that precedes the well known face-sensitive intracranial potential peaking between 160–200-ms [24], [26]–[28], [71]. In these patient studies, category-specificity at this stage could however not be established as a similar early fusiform N110 component was found in response to the presentation of geometrical shapes [71]. In addition, early MEG activity in the occipito-temporal face-selective areas of IOG and FG is compatible with recordings of field potentials and neuronal responses with corresponding early latencies in high-level face-selective patches in the monkey temporal lobe [31], [34], [81]. We also found a face-selective inversion effect in the lateral inferior prefrontal cortex (i.e., posterior IFG / pars opercularis / ventrolateral prefrontal cortex), which is consistent with a small face-selective N110 component from intracerebral recordings in patients [71], and highly face-selective patches in monkey ventral prefrontal cortex [82].

Interestingly, we observed a small early body inversion effect. So far only one EEG study investigated the effect of body inversion on the P1 component [59] but failed to find a significant effect. This is however not in contrast with the present data, as we did not find a body inversion effect at the sensor level, but only after distributed source analysis. Apart from the nonspecific inversion effect in the right medial occipital cortex, early body-selective differential responses were found in the posterio-dorsal medial parietal areas (the precuneus / posterior cingulate), a location consistent with the hemodynamic correlates of perceiving whole body expressions [67] and of visuospatial cognition [83], more specifically mental rotation [84], [85] and passive whole body rotation [86]. Hence, whereas face inversion modulates early activity in face-selective areas in the ventral stream, body inversion evokes differential activity in dorsal stream areas, suggesting different early cortical pathways for face and body perception, and different time courses of activation in the common neural substrate in the fusiform gyrus.

The main location of the early differential activity for houses in present study, the right lateral occipital area, is in agreement with the hemodynamic inversion effect for scenes/houses [87], and more in general with object-processing areas identified with fMRI [88], [89]. The ventral/medial orbitofrontal area agrees with the location of early stimulus categorization found in monkeys [90] and humans using MEG [91].

### Implications for the Neural Mechanisms Underlying Rapid Visual Categorization

Present findings of early category-specific activation of category-sensitive distributed cortical networks between 70–100-ms after stimulus onset are consistent with a growing body of evidence that visual categorization can already take place within the first 100-ms post stimulus onset [32], [33], [92], [93]. These previous studies however mainly provided details on the time-course of visual processing in humans, whereas the question of which anatomical pathways are used to perform such rapid visual categorization still remained a puzzle. It has been hypothesized that, parallel to object- and face-recognition areas in the ventral visual pathway, a subcortical face processing system may exist where a biologically salient stimulus is detected and coarsely processed already before categorical processing in temporal cortex takes place [94], [95]. Alternatively, based on monkey recordings it has been proposed that the same ventral object recognition system that carries out detailed visual analysis at a later stage is also responsible for the initial coarse categorization [34]. The latter would be mediated by a fast feedforward sweep through the ventral stream [32], [96]–[98]. It is not clear however whether the information processing would go further than area V4 or bypass the high-level visual areas of the temporal cortex [32]. For instance, it has been proposed that the low-spatial frequency content of the image is rapidly projected from low-level occipital areas directly to the orbitofrontal cortex within 130-ms by the magnocellular system [91].

The current results provide evidence for the existence of a rapid neocortical route in humans in which information is rapidly transferred from low-level visual areas to high-level category-sensitive visual areas. Our findings suggest that activation started focally in the medial occipital lobe around 50–60-ms, and from here propagated rapidly to more anterior, inferior and lateral areas, extending to the full ventral aspect of the temporal lobe within 100-ms post stimulus onset (Figure 4a). The latencies we found agree with estimates for cortical onset-latencies based on monkey intracranial recordings [76], [99]–[101], and provide support for the notion of a fast feedforward sweep rapidly propagating along a bottom-up hierarchy of ventral areas in which the highest areas are reached within 100-ms [102]. This is further corroborated by the preserved detection of complex naturalistic images by humans and monkeys [31] combined with intact face-selective electrophysiological responses in monkey temporal lobe neurons [31] and hemodynamic responses in human fusiform gyrus [103] under experimental manipulations that disrupt feedback processing but leave the initial feedforward sweep intact (e.g. backward masking [104] and rapid serial visual presentation). Biologically inspired computational models with purely feedforward processing have also been able to account for such rapid categorization [96]–[98].

Direct neurophysiological evidence for the neural mechanisms underlying object categorization in the visual ventral stream stems from monkey recordings, in which face-specific neuronal populations in high-level visual cortex have been found to become activated in two phases [34]. During the first pass (with response onset of 53-ms and average response latency of ca. 90-ms) the neurons coded for rough categorization, e.g., a monkey face or a human face, whereas 50-ms later the same neurons encoded finer information, such as facial identity and emotional expression [34].

Our present MEG findings suggest that similar processes of a first and second pass through the ventral object system may also underlie object recognition in the human brain. The reasons why this first pass has not been fully recognized before may be that during the initial wave of activation sources in low-level occipital areas are far stronger than those in high-level areas, and that the activity in high-level cortical areas is far more prominent during the second wave of activation than during the first pass. To explore the neuronal mechanisms of this subtle first pass in more detail, it would be of interest to investigate whether the early category-specific activation is mediated by the fast magnocellular system which is sensitive to low-spatial frequencies and processes only the coarse information of the image. It has already been shown that low-spatial frequencies only can account for the fast interpretation of natural scenes [105], fast propagation of information about objects to the orbitofrontal cortex [91], the activation of subcortical structures such as the amygdala, pulvinar and superior colliculus by fearful faces [106], emotional modulation of the fusiform face area [107], and for the enhancement of the P1 component to fear-expression [108].

In conclusion, our results provide the first direct evidence of fast category-specific neocortical routes in the human brain, hereby challenging the long-held view that during the first 100-ms only the low-level features of the stimulus are being processed, and that category-specific cortical activation only occurs at later stages.

## Materials and Methods

### Participants

Ten healthy right-handed individuals (mean age 28.4 years, range 24–36 years; four females) with normal or corrected to normal vision volunteered to take part in the experiment. All procedures were approved by the Massachusetts General Hospital Institutional Review Board, and informed written consent was obtained from each participant. The study was performed in accordance with the Declaration of Helsinki.

### Stimuli

Face stimuli were gray-scale photographs from the Ekman and Friesen database [109]. Eight identities (4 male, 4 females) were used, each with a neutral facial expression. Body stimuli were taken from our own validated dataset, previously used in behavioral [62], EEG [64] and fMRI studies [67], [69] and consisted of gray-scale images of whole bodies (4 males, 4 females) adopting a neutral instrumental posture in which the faces were made invisible (for details see [69]). House stimuli were gray-scale photographs taken from eight different real-life brick-stone houses, with prominent orientation cues such as a roof, a door, door steps or part of a sidewalk or garden. Stimuli were processed with photo-editing software in order to equalize contrast, brightness, and average luminance. To create control stimuli that contain the same spatial frequencies, luminance and contrast as their originals, all photographs were phase-scrambled using a two-dimensional Fast Fourier Transform. After randomizing the phases, scrambled images were constructed using the original amplitude spectrum. All images (photographs and scrambles) were pasted into a gray square (with an equal average gray value as the photographs), such that the final size of all stimuli was the same. Examples of the stimulus conditions can be found in Figure 1a.

Since lower half-field stimuli evoke larger visual evoked fields than upper half-field checkerboards [77], possible systematic physical differences between upper and bottom half of the image could potentially confound the results when stimuli are inverted. We therefore checked whether the average luminance for the upper and bottom halves of the images was equal. This appeared to be the case for the Face and Body stimuli. However, for the House stimuli the upper half of the images was found to be significantly brighter than the lower halves.

### Experimental Design

The experiment was divided into four blocks each consisting of 288 trials. Within one block, all stimuli (8 exemplars * 9 stimulus categories) were presented 4 times in random order, summing up to a total of 128 trials for each stimulus condition. Half of the subjects started responding with their left hand, while the other half started with their right hand. At each new block the participants changed the button box to their other hand. To familiarize the subjects with the procedure and task demands the experiment was preceded by a short training session, which contained all stimulus categories.

The experiment was conducted in a magnetically shielded, sound-attenuating room (Imedco AG, Hägendorf, Switzerland). Subjects were comfortably seated with the head leaning against the back of the helmet of the MEG dewar. The visual stimuli were presented with a LP350 Digital Light Processor projector (InFocus, Wilsonville, OR) onto a back-projection screen placed 1.5 m in front of the subject. The size of the framed stimuli on the screen was 17×17 cm, subtending a visual angle of 6.5°. The trial designation is depicted in Figure 1b. The stimuli were presented for 250-ms with an interstimulus interval that ranged between 2500–3000-ms. The stimuli were preceded and followed by a black fixation cross on a gray background, presented for 1000–1500-ms pre-stimulus and 500-ms post-stimulus. The post-stimulus fixation was followed by a screen with the word “PRESS” (1000-ms duration) prompting subjects to make an appropriate button response. The participants' task was to keep their eyes fixed on the cross and to indicate whether the picture was presented Upright, Inverted or Scrambled. In addition, they were instructed to minimize eye blinks and all other movements.

### MEG Data Acquisition

MEG data were acquired with a 306-channel Neuromag VectorView system (Elekta-Neuromag Oy, Helsinki, Finland), which combines the focal sensitivity of 204 first-order planar gradiometers with the widespread sensitivity of 102 magnetometers. Eye movements and blinks were monitored with vertical and horizontal electro-oculogram. The location of the head with respect to the sensors was determined using four head-position indicator coils attached to the scalp. A head-based MEG coordinate frame was established by locating fiduciary landmarks (nasion and preauricular points) with a Fastrak 3D digitizer (Polhemus, Colchester, VT). The data were digitized at 600 samples/second with an anti-aliasing low-pass filter set at 200 Hz.

MEG signals were averaged across trials for each condition, time-locked to the onset of the stimulus. A 34-ms delay between the time the computer sent an image and the time it was projected onto the screen was measured with a photodiode and subsequently taken into account when reporting the timing of measured activity. A 200-ms pre-stimulus period served as baseline. Trials to which subjects made an incorrect response and those that contained eye blinks exceeding 150 µV in peak-to-peak amplitude or other artifacts were discarded from the average. The evoked responses were low-pass filtered at 40 Hz.

### Structural magnetic resonance imaging (MRI)

MEG data were co-registered with structural high-resolution magnetic resonance images (MRI). A set of 3-D T1-weighted MR images using a 1.5 T system were acquired. The MRI and MEG coordinate systems were aligned by identifying the fiducial point locations in the MRIs. In addition several points were digitized from the head surface to allow confirmation and fine tuning of the initial alignment based on the fiducial landmarks.

The geometry of the cortical mantle was extracted from the MRI data using the Freesurfer software [110], [111]. An inflated representation of the cortical surface was used for visualization to allow viewing the gyral pattern and the cortex embedded in fissures.

### Global MEG measures

MEG data was first quantified at the sensor level. The mean global field power (MGFP) was calculated separately for the magnetometers and the gradiometers by squaring the signal values and averaging them across sensors. Another global measure was obtained by averaging the time courses of the estimated currents across all dipoles (see next section) in each individual brain. Statistical group analysis was performed by two-tailed *t*-tests for paired samples (*α* = 0.05) on the MGFP and the mean current strength across dipoles respectively at successive time points.

### MEG Source Estimation

The source current distribution was estimated at each cortical location using the minimum-norm estimate (MNE) [112]. The cortical surface was sampled with ca. 5000–7000 dipoles at the interface between gray and white matter provided by Freesurfer with an average 7-mm spacing between adjacent source locations. The forward solution for each of the three dipole components at each of these locations was computed for all 306 sensors using an anatomically realistic single-layer Boundary Element Model [113]. The inner skull boundary for this model was derived from each subject's MRI. The strength of the fixed-location sources was estimated for each time-instant of the evoked response applying the linear inverse solution using a cortical loose orientation constraint [114]. The resulting current amplitudes were noise-normalized by dividing the magnitude of the estimated currents at each location by their respective standard deviations [70]. The latter was estimated with help of the spatial noise-covariance matrix, which was computed from the 200-ms pre-stimulus activity in the non-averaged data set with the same filter settings as for the evoked responses. This noise-normalization procedure reduces the location bias towards superficial currents, inherent in the minimum-norm solution, and equalizes the point-spread function across cortical locations [70]. The noise-normalized solution provides a dynamical Statistical Parametric Maps (dSPM), which essentially indicates the signal-to-noise ratio of the current estimate at each cortical location as a function of time. Thus, dSPM movies of brain activity are useful for visualization of the data as they identify locations where the MNE amplitudes are above the noise level.

Group movies were created by morphing the source estimates for each individual subject to the cortex of one representative subject, according to the method of Fischl et al. [115]. Subsequently, the values were averaged across individuals at each source location. The dSPM values were used to identify spatiotemporal cortical patterns that show consistent responses across individuals. To quantify the Inversion Effects the actual source amplitudes of the MNE were used in parametric statistical testing rather than the dSPM values. Two-tailed paired *t*-tests (*df* = 8, *n* = 9, *α* = 0.01) were performed between the Upright and Inverted conditions for each dipole location and each time point. The results were thresholded for the baseline noise, i.e. significant *t*-values at the level of *p*<0.01 were visualized only if the currents exceeded a signal-to-noise ratio of 2.5 (dSPM>2.5) in at least one of the two stimulus conditions. Next, the source distributions of the category-specific M100 Inversion Effects were determined by taking the largest significant (supra-baseline-noise) positive or negative t-values at each dipole location occurring within the 70–100-ms time-window.

### Spatial overlap in MNE maps

The amount of spatial overlap between the category-specific M100 Inversion Effects was quantified by calculating the number of dipoles that met the M100 Inversion Effect criteria described above for one, two or three of the categories.

### MNE time courses extracted from selected regions

To investigate the time courses of the main regions that showed significant group Inversion Effects in the average brain in more detail, the corresponding anatomical regions were drawn in the reconstructed inflated cortical surface of each individual. The regions selected were the calcarine sulcus, Inferior Occipital Gyrus (IOG), Lateral Occipital Cortex (LOC), the middle part of the Fusiform Gyrus (mFG), the transition region of the posterior Inferior Frontal Gyrus (pIFG; pars opercularis) and middle part of the superior circular insular sulcus, and the posterio-dorsal medial parietal areas (Precuneus and the posterior Cingulate Gyrus). For the region of the calcarine sulcus, we excluded the anterior half of the calcarine fissure, representing peripheral visual field eccentricities that were not stimulated in our protocol, and selected the posterior half of the calcarine fissure including a small strip (ca. 2 mm) of the adjacent gyri. The fusiform gyrus (FG) was divided into three parts along its anteroposterior axis, resulting into an anterior, middle and posterior part. The LOC comprised the anatomical regions of the middle occipital gyrus and sulcus and the anterior occipital sulcus. The time courses of the estimated currents for each dipole within these selected regions were extracted and used for further analysis. Two-tailed *t*-tests for paired samples (*α* = 0.01) were performed on the mean current strength across dipoles at successive time points.
